# Kikuchi’s disease

**DOI:** 10.4103/0973-029X.64300

**Published:** 2010

**Authors:** Babu N Chaitanya, CS Sindura

**Affiliations:** *Department of Oral Pathology, M.S. Ramaiah Dental College and Hospital, MSRIT Post, New BEL Road, Bangalore - 560 054, Karnataka, India*

**Keywords:** Kikuchi’s disease, lymphadenitis, lymphoma, systemic lupus erythematosus

## Abstract

Kikuchi disease is an idiopathic, generally self-limiting cause for lymphadenitis that can be clinically and histologically mistaken for lymphoma or systemic lupus erythematosus. Differentiating this disease from common lymphatic disorder is extremely important from the pathologist’s point of view, which is highlighted in the article.

## INTRODUCTION

The disease was first described in Japan in 1972. More recently, the disease has been reported throughout the world and in all races.[1]

## ETIOLOGY

There is much speculation about the etiology of Kikuchi’s disease, although infectious and autoimmune causes have been suggested. The most favored theory is that Kikuchi disease results when one or more unidentified agents trigger a self-limited autoimmune process. It is proposed that Kikuchi’s disease is a nonspecific hyperimmune reaction to a variety of infectious, chemical, physical and neoplastic agents. Lymphadenitis results from apoptotic cell death induced by cytotoxic T lymphocytes. Human leukocyte antigen class II genes are more frequent in patients with Kikuchi disease, suggesting a genetic predisposition to the proposed autoimmune response.[1]

Features that support a role for an infectious agent include upper respiratory tract infections and several viral infections caused by cytomegalovirus, Epstein-Barr virus,[2] human herpesvirus, varicella-zoster virus, parainfluenza virus, parvovirus B19 and paramyxovirus.[1]

Several authors have reported an association between Kikuchi disease and systemic lupus erythematosus (SLE). Kikuchi disease has been diagnosed before, during and after a diagnosis of SLE is made in the same patient. Additionally, the histologic appearance of lymph nodes in patients with Kikuchi disease is similar to that of patients with SLE lymphadenitis. But, however, association of Kikuchi disease with SLE, if any, remains unclear.[1]

## CLINICAL FEATURES

Kikuchi disease has been reported throughout the world and in all races.[1] Women are affected more often than men, by a ratio of approximately 3:1. But, recent reports suggest the ratio to be 1:1. Kikuchi disease occurs in a wide age range of patients (i.e., 2–75 years), but it typically affects young adults (mean age, 20–30 years).[1]

This disease most frequently manifests as a relatively acute onset of cervical adenopathy associated with fever and a flu-like prodrome.[1] The most common clinical manifestation is cervical lymphadenopathy with or without systemic signs and symptoms.[3–6]

- Lymphadenopathy
  Cervical nodes are affected in about 80% of the cases. Posterior cervical nodes are frequently involved (65–70% of the cases). Lymphadenopathy is isolated to a single location in 83% of the cases, but multiple chains may be involved. The nodes are usually described as painless or mildly tender. The nodes that are usually firm and mobile tend to be 2-3 cm in diameter, although masses of multiple nodes may reach 6 cm.[1]
- Flu-like prodrome with fever is present in 50% of the cases. Less common symptoms include:
  Headache, nausea, vomiting, malaise, fatigue, weight loss, arthralgias, myalgias, night sweats, rash, thoracic/abdominal pain.[1]

## EXTRANODAL FINDINGS

The incidence of skin involvement varies from 5 to 30%. Findings are nonspecific, which include maculopapular lesions, morbilliform rash, nodules, urticaria and malar rash, which may resemble that of SLE.[7,8] Although neurologic involvement is rare, conditions like aseptic meningitis, acute cerebellar ataxia and encephalitis can occur[9] Widespread involvement of multiple organ systems has been described in solid-organ transplant patients.[1]

## DIFFERENTIAL DIAGNOSIS OF KIKUCHI DISEASE FROM A PATHOLOGIST’S POINT OF VIEW

- SLE
- Lymphomas
- Tuberculosis (TB)
- Infectious mononucleosis
- Syphilis
- Sarcoidosis
- Adenocarcinomas[1]

## LABORATORY INVESTIGATIONS

- Complete blood cell count
  Mild granulocytopenia is observed in 20–50% of the patients. Leukocytosis is present in 2–5% of the patients. Atypical lymphocytes are observed in 25% of the patients.
- Erythrocyte sedimentation rate and C-reactive protein levels may be elevated.[1]
- An elevated lactate dehydrogenase level suggests hepatic involvement.[1]

## IMAGING STUDIES

Diagnostic imaging studies confirm the presence of enlarged lymph nodes in the affected areas, but they cannot specifically confirm the diagnosis of Kikuchi disease.[1]

Computed tomography scan, magnetic resonance imaging and ultrasonography are used to confirm the presence of lymph nodes.[10]

## LAB PROCEDURES

### Fine needle aspiration cytology

A definitive diagnosis of Kikuchi disease can be made only by tissue evaluation. Cytologic examination by fine needle aspiration cytology (FNAC) can suggest the diagnosis of Kikuchi disease, when supported by typical clinical findings,[11,12] but excisional biopsy of an involved lymph node is needed to confirm the diagnosis in doubtful cases.[1]

In a retrospective study of 44 patients, FNAC achieved an accuracy of 56.75% in diagnosing Kikuchi disease[13] Characteristic cytologic findings include crescentic histiocytes, plasmacytoid monocytes and extracellular debris.[1] Pathologists are likely to report the result as “suggestive of” Kikuchi disease.

It is therefore advisable to confirm the diagnosis of Kikuchi’s disease by excisional biopsy in doubtful cases.[1]

### Excisional lymph node biopsy

Histologic findings consistent with Kikuchi disease include:

- Paracortical necrosis, which may be patchy or confluent, the degree of necrosis varying considerably from one case to another.
- Histiocytes, crescent-shaped nuclei (crescentic nuclei), karyorrhexis, histiocytes and macrophages containing phagocytozed debris from degenerated lymphocytes are seen
- Other cells include lymphocytes, plasmacytoid monocytes, macrophages and immunoblasts (predominantly T cells).[1]

## HISTOLOGIC FINDINGS

The three histological phases of Kikuchi disease are[6]

- Proliferative phase: initial phase with typical findings noted above
- Necrotizing phase: extensive necrosis that may destroy the normal architecture of the lymph node
- Xanthomatous (foamy cell) phase: the recovery phase with resolution of necrosis

## TRANSMISSION ELECTRON MICROSCOPY STUDIES

Transmission electron microscopy (TEM) from lymph nodes revealed specific morphological features of apoptotic cells such as nuclear chromatin condensation and fragmentation along the nuclear membrane with intact organelles, presence of histiocytes, phagocytosing karyorrhectic debris (apoptotic bodies) in areas affected by Kikuchi’s disease.[14]

## IMMUNOHISTOCHEMICAL STUDIES

- The immunophenotype of Kikuchi disease is primarily composed of mature CD8-positive and CD4-positive T lymphocytes.
- Positive immunostaining results by monoclonal antibody Ki-M1P is seen in Kikuchi disease.[1]
- The immunostain with HECA 452 (directed against cutaneous lymphocyte antigen) highlighted numerous transformed lymphocytes and plasmacytoid monocytes. The later along with macrophages also expressed PG-M1 against the macrophage-restricted CD 68 epitope.[15]

## DISCUSSION

Kikuchi disease, also known as apoptotic lymphadenitis or histiocytic necrotizing lymphadenitis, is a benign, self-limiting condition of unknown etiology.

Clinically and histologically, this disease can be mistaken for lymphoma or SLE.[2–6,16,17] Hence, differentiating this from common lymphadenopathic conditions is extremely vital.

Dorfman stated that although the differential diagnosis may include several nonneoplastic conditions such as SLE, toxoplasmic lymphadenitis, infectious mononucleosis and cat-scratch disease, the main diagnostic problem encountered by the histopathologist is to distinguish Kikuchi disease from non-Hodgkin’s lymphoma.[18]

It is very important to distinguish Kikuchi s disease from these nonneoplastic conditions and other lymphatic disorders because the course and treatment differ dramatically for each.

### Distinguishing Kikuchi disease from lymphoma

Malignant lymphoma is the most important differential diagnosis in both clinical and histological terms.

Malignant lymphoma, especially T-cell non-Hodgkin’s lymphoma, can be mistaken for Kikuchi’s disease. Loss of T-cell antigens by immunostains and determination of the monoclonality of T cells by molecular studies is necessary for confirming the diagnosis of T-cell lymphoma.[1]

- The numerous atypical monocytes and T-cell immunoblasts observed in Kikuchi disease may lead to an erroneous diagnosis of lymphoma, especially high-grade lymphoma. However, lymphoma typically features cytologic atypia and monomorphic cells. Features of Kikuchi disease that may help prevent its misdiagnosis as malignant lymphoma include incomplete architectural effacement with patent sinuses, presence of numerous reactive histiocytes, relatively low mitotic rates and absence of Reed-Sternberg cells.[1]
- Immunohistochemical stains are helpful in distinguishing Kikuchi s disease from lymphomas. The large cells are negative for CD3, CD20, which excludes the possibility of lymphoma, and they are positive for CD68, which demonstrates their histiocytic feature.[19] Positive immunostaining results by monoclonal antibody Ki-M1P are seen in Kikuchi disease but not in malignant lymphoma.

### Distinguishing Kikuchi disease from SLE

- Kikuchi disease and SLE have similar histopathologic appearances. Kikuchi disease is suggested by the absence or paucity of hematoxylin bodies, plasma cells and neutrophils.[1]
- In SLE, the enlarged lymph nodes may also contain areas of necrosis and karyorrhexis, but they typically show follicular hyperplasia with an abundance of granulocytes and plasma cells. Kikuchi’s disease is associated with proliferation of mitotically active, large blastic cells made up of a mixture of T lymphocytes and heterogenous proliferation of histiocytes.[19]

### Differentiating Kikuchi disease from infectious mononucleosis

The diagnosis of infectious mononucleosis is made on the basis of characteristic clinical, hematological and serological findings.[20]

### Differentiating Kikuchi disease from TB

Biopsy of lymph nodes with extensive areas of necrosis should be interpreted very carefully because TB is the most common cause of lymph node necrosis. However, classic features of Kikuchi’s disease will be sufficient to avoid misdiagnosis with TB in the majority of the cases.[20]

## CONCLUSION

Symptoms of Kikuchi’s disease can be very distressing to the patient, especially the lingering fever and fatigue. It is important for pathologists and clinicians to be aware of this possibility, especially when dealing with young female patient with fever and cervical lymphadenopathy.

Early recognition of Kikuchi’s disease will minimize potentially harmful and unnecessary evaluations and thus prevent misdiagnosis and inappropriate treatment. We can thereby avoid laborious investigations for infectious and lymphoproliferative disorders. Hope this review will spawn further studies in this regard that help in more therapeutic intervention that will be beneficial for both the patient and the treatment provider.
